# Ups and downs in the search for a Herpes simplex virus vaccine

**DOI:** 10.7554/eLife.06883

**Published:** 2015-03-10

**Authors:** Silvia Bolland, Susan K Pierce

**Affiliations:** Laboratory of Immunogenetics, National Institute of Allergy and Infectious Diseases, Rockville, United States; Laboratory of Immunogenetics, National Institute of Allergy and Infectious Diseases, Rockville, United Statesspierce@niaid.nih.gov

**Keywords:** vaccine, HSV-2, virology, antibody, immunity, mouse

## Abstract

Modified herpes simplex viruses that are unable to produce glycoprotein D may make effective vaccines.

**Related research article** Petro C, González PA, Cheshenko N, Jandl T, Khajoueinejad N, Bénard A, Sengupta M, Herold BC, Jacobs WR Jr. 2015. Herpes simplex type 2 virus deleted in glycoprotein D protects against vaginal, skin and neural disease. *eLife* **4**:e06054. doi: 10.7554/elife.06054

**Image** A cell infected with the herpes simplex virus

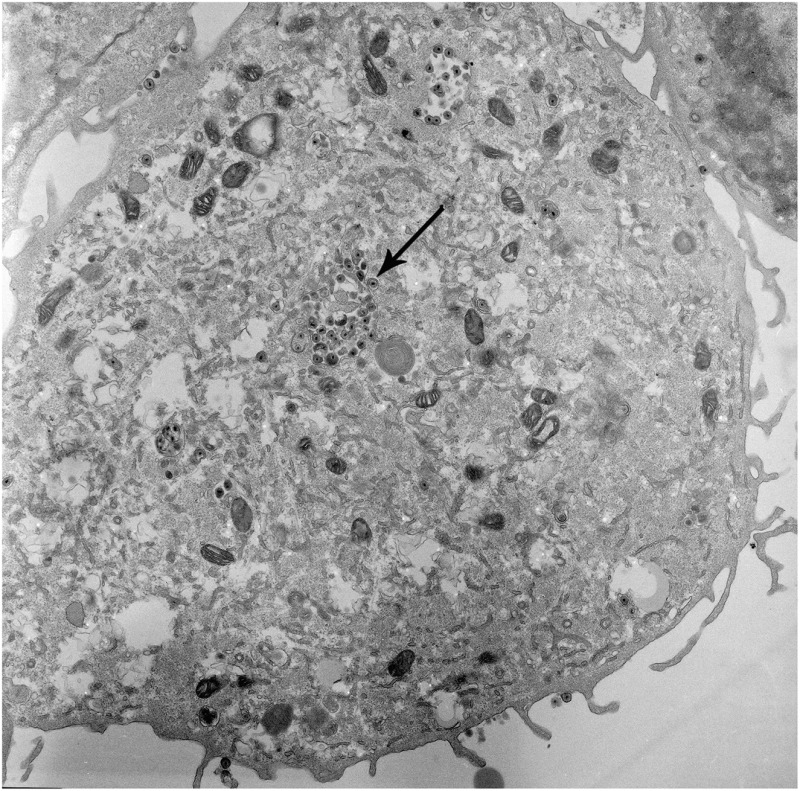

Herpes simplex virus (HSV) infections impose an enormous health burden on the world's population, making the development of an HSV vaccine a top public health priority (Shin and Iwasaki, 2013; Awasthi and Friedman, 2014). HSV-1 is the leading cause of corneal blindness worldwide and has emerged as the predominant cause of genital disease in the developed world. HSV-2 is the leading cause of genital ulcerative disease, and its high prevalence in sub-Saharan Africa contributes significantly to the spread of HIV-1 in this region. Moreover, HSV can lie dormant (or latent) in neurons for months or years before becoming active, so the health of infected individuals can be affected for life. There is, therefore, an urgent need for an effective HSV vaccine that can provide protection against infection and also prevent the virus entering a latent state. Now, in *eLife*, William Jacobs and Betsy Herold of the Albert Einstein College of Medicine and co-workers—including Christopher Petro and Pablo González as joint first authors—have taken a giant step toward meeting that need (Petro et al., 2015).

For nearly 20 years, HSV-2 glycoprotein D, which makes up part of the outer coating of the virus and is required for HSV infection of cells, has been the predominant HSV vaccine candidate (Shin and Iwasaki, 2013; Awasthi and Friedman, 2014). HSV infection stimulates the immune system to produce predominantly glycoprotein D-specific antibodies: these neutralizing antibodies bind to the virus and block its ability to infect cells. Unfortunately, thus far the outcomes of clinical trials of vaccines based on HSV-2 glycoprotein D have been disappointing (Belshe et al., 2012). Now, somewhat counterintuitively, Petro, González et al. have developed a potential vaccine using an HSV-2 virus that lacks the gene that encodes glycoprotein D.

Because HSV-2 requires glycoprotein D to infect cells, Petro, González et al. produced their vaccine by growing a glycoprotein D-deficient virus in a culture of cells that expressed the HSV-1 form of glycoprotein D. This allowed the viruses to capture glycoprotein D from the cells and replicate. The resulting virus (referred to as HSV-2 ΔgD^−/+D−1^) was predicted to be safe, as it should be able to infect mouse cells but not be able to produce infectious progeny. Importantly, there was no evidence that this method created recombinant viruses that gained the gene encoding HSV-1 glycoprotein D, which would have allowed the virus to establish a deadly infection in vaccinated mice.

Vaccinating mice with the modified HSV-2 ΔgD^−/+D−1^ virus was proven to be both safe and effective. The vaccine did not cause disease in severely immune-deficient mice, and it prevented vaginal, skin and neuronal disease, and also blocked latency, in two different mouse strains. Furthermore, the antibodies generated by the mice in response to the vaccination were sufficient to transfer protection to unimmunized mice, but only if the immune cells of the recipient mice expressed FcγR. FcγR is a receptor that binds to antibodies attached to virus-infected cells and triggers the immune cells to kill the infected cell, a phenomenon termed antibody-dependent cellular cytotoxicity (ADCC). Indeed, Petro, González et al. demonstrated that the antibodies from vaccinated mice induced ADCC of virus-infected cells in vitro.

This is all very good news for HSV vaccine development, but also presents a cautionary message that could guide future HSV vaccine design. Previous vaccine development focused on HSV-2 glycoprotein D in part because of its ability to stimulate the production of neutralizing antibodies. But Petro, González et al. have now shown that HSV-specific antibodies with only low levels of neutralizing activity were highly effective in transferring protection to non-vaccinated mice, presumably via ADCC.

These findings raise some questions: are neutralizing antibodies that are specific for HSV-2 glycoprotein D unable to trigger antibody-dependent cellular cytotoxicity and does the expression of HSV-2 glycoprotein D in itself dampen ADCC? This form of cytotoxicity is mediated in large part by immune cells called natural killer cells. Natural killer cells recognize cells targeted for killing by ADCC because target cells display ligand molecules on their surface that interact with a variety of natural killer cell-activating receptors, including Fc receptors (Long et al., 2013). It is possible that HSV-2 glycoprotein D-specific neutralizing antibodies bind avidly to the virus but only poorly to glycoprotein D expressed on virus-infected cells, and thus are weak inducers of natural killer cell ADCC (Figure 1). In contrast, in the absence of HSV-2 glycoprotein D, mice mount antibody responses to a variety of HSV-2 proteins that are not highly neutralizing but together may induce strong natural killer cell ADCC of infected cells. In addition, the inherent properties of HSV-2 glycoprotein D itself may contribute to weak ADCC. For example, it was recently shown that the expression of HSV-2 glycoprotein D reduced the levels of a molecule called CD122 on infected cells, which reduced the ability of natural killer cells to kill the virus-infected cells (Grauwet et al., 2014).

**Figure 1.**
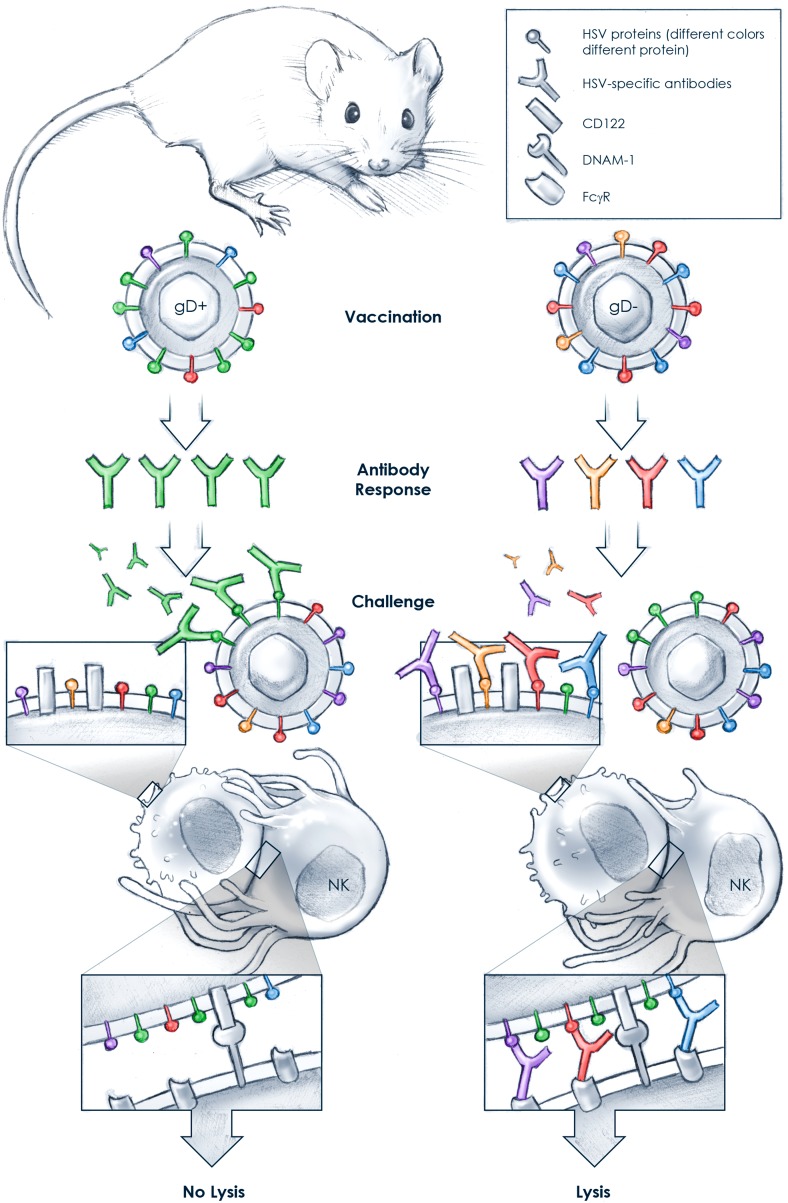
Why vaccination with a glycoprotein D-deficient Herpes simplex virus (HSV) may induce protection. Natural killer (NK) cells have many receptors, at least two of which—FcγR and DNAM-1—recognize antibody-decorated, virus-infected target cells: the antibodies bound to the infected cells are detected through FcγR, and a protein called CD122 on the surface of the infected cells is detected through DNAM-1. Vaccination with a glycoprotein D-expressing virus (gD^+^; left) induces predominantly neutralizing antibodies specific for glycoprotein D (green). Upon challenge with HSV, these antibodies bind strongly to the virus but may not bind well to the surface of the virus-infected cell. Consequently, the NK cell FcγRs are not engaged and binding of the NK cell's DNAM-1 to CD122 is not sufficient to induce the killing (by lysis) of the infected cell. In contrast, Petro, González et al. found that vaccination with a gD-deficient virus (gD^−^; right) induces the production of primarily non-neutralizing antibodies that are specific for a variety of glycoproteins on the surface of the virus. The antibodies in the gD^−^-vaccinated mouse bind poorly to HSV but strongly to the surface of the infected cells. The NK cells can therefore detect the infected cells through both FcγR and DNAM-1, which is sufficient to activate the NK cells to kill the target cells by lysis.

Why glycoprotein D is not an effective vaccine and why the removal of glycoprotein D from HSV-2 makes an effective vaccine is a puzzle that we may not solve soon. Nevertheless, it is indeed a good day in the struggle to develop a vaccine to protect the world's population against the disease burden of HSV infections!
